# Retraction: Protein Isoaspartate Methyltransferase Prevents Apoptosis Induced by Oxidative Stress in Endothelial Cells: Role of Bcl-X_l_ Deamidation and Methylation

**DOI:** 10.1371/journal.pone.0207530

**Published:** 2018-11-08

**Authors:** The PLOS ONE Editors

After publication of this article [1], concerns were raised about the data presented in Fig 3B. Specifically, it was noted that lanes 1–3 of the PARP blots shown for Control and Sense experiments appear highly similar, although with slightly different aspect ratios and an additional 89 kDa band in lane 3 of the Control blot. Similarities were also noted between lanes 1 and 2 of the Caspase blots for Control and Sense experiments.

The first author noted that the primary data underlying the results in Fig 3 are no longer available.

In light of these issues, the *PLOS ONE* Editors retract this article due to concerns about the reliability and integrity of the results.

RC, MLDB did not respond. AC, FB, IS, SD, DI did not agree with retraction. LM, VZ, PG, could not be reached.
